# The recognition mode between hsRBFA and mitoribosome 12S rRNA during mitoribosomal biogenesis

**DOI:** 10.1093/nar/gkac1234

**Published:** 2023-01-09

**Authors:** Wanwan Zhou, Xiaodan Liu, Mengqi Lv, Yunyu Shi, Liang Zhang

**Affiliations:** Hefei National Laboratory for Physical Sciences at the Microscale, School of Life Sciences, Division of Life Sciences and Medicine, University of Science and Technology of China, Hefei, Anhui 230027, P.R. China; Ministry of Education Key Laboratory for Membraneless Organelles and Cellular Dynamics, University of Science & Technology of China, Hefei, P.R. China; Hefei National Laboratory for Physical Sciences at the Microscale, School of Life Sciences, Division of Life Sciences and Medicine, University of Science and Technology of China, Hefei, Anhui 230027, P.R. China; Ministry of Education Key Laboratory for Membraneless Organelles and Cellular Dynamics, University of Science & Technology of China, Hefei, P.R. China; Hefei National Laboratory for Physical Sciences at the Microscale, School of Life Sciences, Division of Life Sciences and Medicine, University of Science and Technology of China, Hefei, Anhui 230027, P.R. China; Ministry of Education Key Laboratory for Membraneless Organelles and Cellular Dynamics, University of Science & Technology of China, Hefei, P.R. China; Hefei National Laboratory for Physical Sciences at the Microscale, School of Life Sciences, Division of Life Sciences and Medicine, University of Science and Technology of China, Hefei, Anhui 230027, P.R. China; Ministry of Education Key Laboratory for Membraneless Organelles and Cellular Dynamics, University of Science & Technology of China, Hefei, P.R. China; Hefei National Laboratory for Physical Sciences at the Microscale, School of Life Sciences, Division of Life Sciences and Medicine, University of Science and Technology of China, Hefei, Anhui 230027, P.R. China; Ministry of Education Key Laboratory for Membraneless Organelles and Cellular Dynamics, University of Science & Technology of China, Hefei, P.R. China

## Abstract

Eukaryotes contain two sets of genomes: the nuclear genome and the mitochondrial genome. The mitochondrial genome transcripts 13 mRNAs that encode 13 essential proteins for the oxidative phosphorylation complex, 2 rRNAs (12s rRNA and 16s rRNA), and 22 tRNAs. The proper assembly and maturation of the mitochondrial ribosome (mitoribosome) are critical for the translation of the 13 key proteins and the function of the mitochondrion. Human ribosome-binding factor A (hsRBFA) is a mitoribosome assembly factor that binds with helix 28, helix 44 and helix 45 of 12S rRNA and facilitates the transcriptional modification of 12S rRNA during the mitoribosomal biogenesis. Previous research mentioned that the malfunction of hsRBFA will induce the instability of mitoribosomes and affect the function of mitochondria, but the mechanisms underlying the interaction between hsRBFA and 12S rRNA and its influence on mitochondrial function are still unknown. In this study, we found that hsRBFA binds with double strain RNA (dsRNA) through its whole N-terminus (Nt) instead of the KH-like domain alone, which is different from the other homologous. Furthermore, we mapped the key residues that affected the RNA binding and maturation of mitoribosomes *in vitro*. Finally, we investigated how these residues affect mitochondrial functions in detail and systematically.

## INTRODUCTION

Mitochondria are indispensable organelles in eukaryotic cells. Their main function is to produce ATP through the oxidative phosphorylation process (OXPHOS), which provides energy for the routine life activities of cells (1–5). Mitochondria have a unique genome (mt-DNA) and mitochondrial transcription machinery and mitochondrial translation machinery (2,6,7). In a mammalian cell, the mitochondrial ribosome, which is composed of the 28S small subunit and 39S large subunit, translates 13 intimal proteins that are key components of the oxidative phosphorylation super complex (8–11). Both small and large subunits contain one rRNA and numerous proteins and depend on the coordination of multiple modification steps before their proper assembly (12–15).

In the process of mitochondrial ribosome synthesis, ribosomal RNA posttranscriptional modification is involved in transcription, splicing, translation, ribosomal maturation and function (13,16–21). hsRBFA is a mitochondrial ribosomal binding factor. Studies have found that it mainly binds to the regions including the 44th and 45th cervical ring structures (helix 44, helix 45) of the mitochondrial small subunit 12S ribosomal RNA (22,23) in human. hsRBFA can help TFB1M complete the dimethylation modification of helix 45 to promote the maturation of the small subunit (22,24). This special modification is considered a marker of normal assembly of the mitochondrial ribosomal subunits in eukaryotes. Knockdown of hsRBFA resulted in a decrease in the dimethylation level of 12S rRNA (22). In addition, other studies found that hsRBFA interacts with another methyltransferase, METTL15, which catalyzes the methylation of helix 44 of 12S rRNA (25,26). These results suggest that hsRBFA plays an important role in multiple methylating modifications of 12S rRNA of the mitochondrial small subunit.

**Figure 1.**
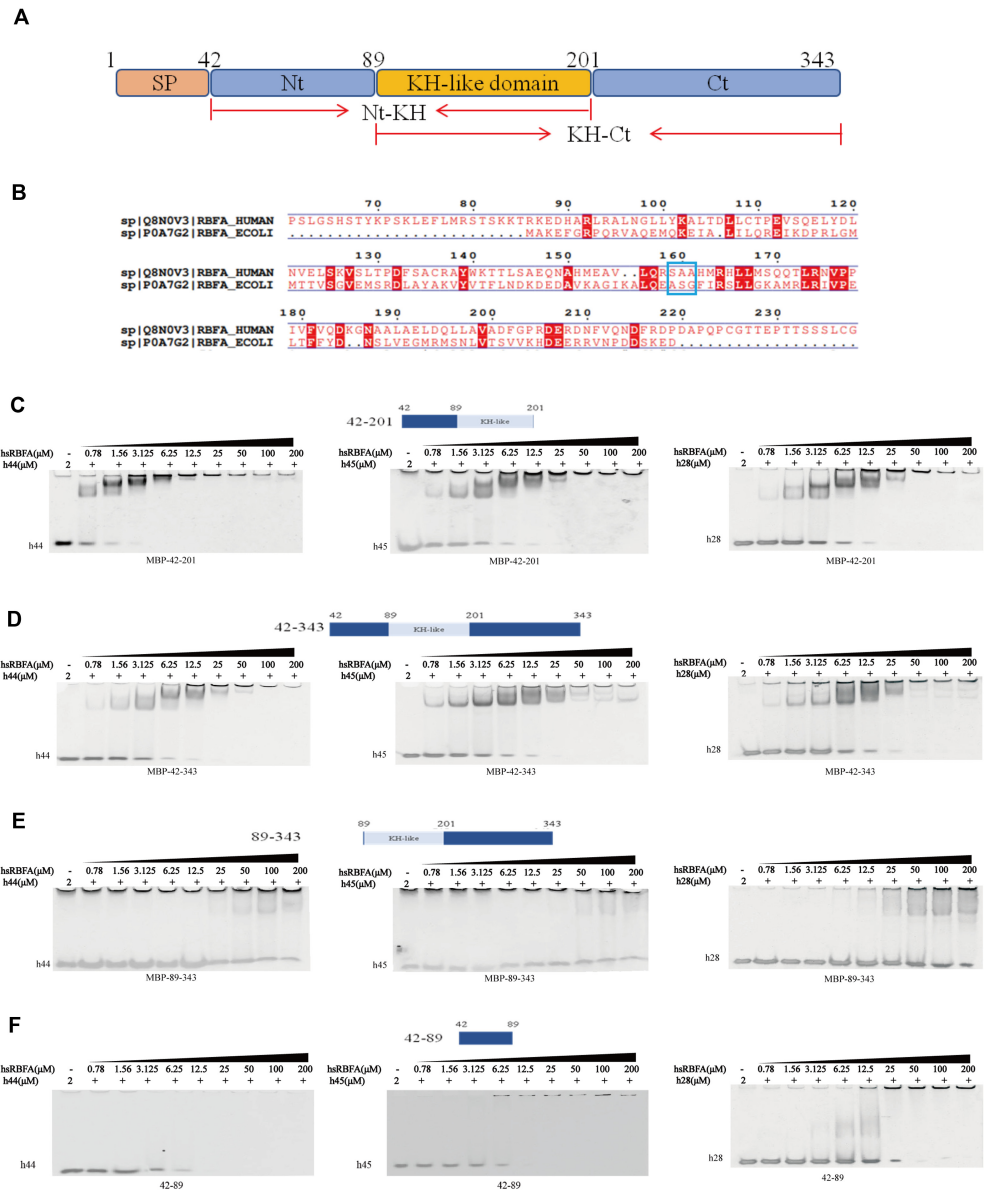
hsRBFA interacts with 12S rRNA through its N-terminus and KH-like domain. (**A**) Schematic diagram illustrating the domain organization of hsRBFA. (**B**) Pairwise sequence alignment of KH-like domains of hsRBFA and RBFA from *E coli*. The blue square marks the AxG motif. (**C–F**) EMSA of the affinity between helix 44, helix 45 or helix 28 of 12S rRNA and different truncated mutations of hsRBFA.

In terms of evolution, hsRBFA has low sequence similarity with its prokaryotic homologous protein (16% similarity), but hsRBFA contains a conserved KH-like domain (22,27) (Figure 1A). The KH domain (K homology domain) is a classical RNA or single-strand DNA binding domain with two different types of folding (28–32). Type I domains are found in multiple copies in eukaryotic proteins, whereas type II KH domains are typically found as single copies in prokaryotic proteins (29,33,34). For both types, the primary interaction site is an interface formed by the two α-helices in the minimal fold motif. The α-helices are connected by a Gly-x-x-Gly (GxxG) sequence, which is a conserved signature motif common to many type I or type II domains (35–37). The KH-like domain of hsRBFA belongs to type II KH folding. However, the GxxG motif is replaced by Ala-x-Gly, which is characteristic of prokaryotic RBFA homologs (38,39). Interestingly, the Ala-x-Gly (AxG) motif is apparently missing in hsRBFA (27) (Figure 1B). RBFA is also a cold shock protein in bacteria and is necessary for cell growth at low temperatures (40–43). It can bind with IF3 to complete ribosomal biogenesis (assisting in the shearing of 17S rRNA) and translation initiation and improve tolerance (44). The assembly of small ribosomal subunits can also promote the association of RsgA (a GTPase that is necessary for ribosome assembly) with the 30S subunit (45). In a study of the ribosome cryo-EM structure of *thermophilic bacteria*, RBFA was found to be located on the small subunit of the ribosome, in contact with three interfaces, and at the tip of 16S rRNA helix 44 and helix 45 (46). However, the resolution is too low (12 Å) to elucidate the binding mechanism. Another study in *Escherichia coli* showed that RBFA is involved in the formation of the central pseudoknot and docking of helix 44 to the decoding center (47). The complex structure of RBFA binding with the *E. coli* small ribosomal subunit was also reported recently. In this work, the *E. coli* RBFA contacts the 3′ end of 16S rRNA through the classic KH combination mode (21).

hsRBFA binds to the small subunit of mitoribosomes, but it differs in the binding site in rRNA from other RBFAs reported. hsRBFA binds to helix 44, helix 45, and helix 28 of the 12S rRNA in the small subunit, yet the detailed location or manner is unclear (22,23,48). Although a recent cryo-electron microscopy study on the structure of the mitochondrial ribosomal small subunit (SSU) suggested that RBFA interacts with helix 28 and the 3′ end of 12S rRNA in the early stages of SSU assembly in mouse, there is no clear evidence, especially the molecular details for this in human are unknown (23). During the maturation of the small subunit, hsRBFA supported TFB1M and METTL15 to catalyze the demethylation and methylation of 12S rRNA sequentially and promoted the assembly of the mitochondrial ribosome by allowing the initiation factor (mtIF3) to bind (49). Finally, the replacement of hsRBFA by mS37 will complete the assembly of the SSU–mS37–mtIF3 complex and initial translation (23). However, in their study, the binding interfaces between hsRBFA and RNAs, particularly the N-terminus of hsRBFA, were not clear. Therefore, in our study, we comprehensively provided a new binding model of hsRBFA to its substrate in the 12S rRNA: the binding of hsRBFA to 12S rRNA was not dependent on its KH-like domain but on the basic amino acid of its N-terminal. Meanwhile, hsRBFA is bound to double-stranded RNA instead of single-stranded RNA, as previously predicted. Additionally, we further investigated the mechanism by which hsRBFA systematically and in detail affects mitochondrial functions in cells. Taken together, our work provides a new way to study the binding between the KH-like protein and its substrate.

## MATERIALS AND METHODS

### Cloning, expression and purification of hsRBFA

The hsRBFA open reading frame was amplified using PCR from a cDNA library constructed from the human brain. The amplified fragment was cloned into the modified pET-22b, which contained N-terminal His6 and maltose-binding protein (MBP) tags. The His6-tagged MBP-hsRBFA fused protein was expressed in *E. coli* strain BL21-Gold (DE3) cells. The cells were grown to mid-log phase (OD600, 0.8–1.0) in Luria Broth containing 50 μg/ml ampicillin at 37°C. The overexpression of the fusion protein was induced using 0.5 mM isopropyl-d-thiogalactopyranoside (IPTG) for 24 h at 16°C, after which the cells were collected by centrifugation. Cells paste from 1 l of culture was suspended in 40 ml of precooled buffer A (20 mM Tris, pH 7.5, containing 1 M sodium chloride). An FB-110X homogenizer was used to lyse the cells at a pressure of 800 bar. The cells were subsequently lysed by sonication on ice to remove nonspecific-binding nucleic acids and centrifuged at 10 000g for 30 min. The protein-containing supernatant was subjected to affinity chromatography using a Ni2+-chelating column (GE Healthcare), eluted with precooled buffer B (20 mM Tris, pH 7.5, containing 1 M sodium chloride and 0.5 M imidazole) and purified by size-exclusion chromatography using a Superdex 200 column (GE Healthcare). The purified hsRBFA protein was diluted in buffer C (20 mM Tris pH 7.5, containing 200 mM NaCl).

### Electrophoresis mobility shift assay (EMSA)

EMSA was used to measure the binding affinities of wild-type (WT) and mutant hsRBFA for RNA (RNA sequences shown in Supplementary Table S1). Experiments were performed in 10 μl volumes of buffer D (20 mM Tris–HCl, pH 8.0, containing 200 mM sodium chloride) containing 200 nM ssRNA or 2μM dsRNA and increasing concentrations of hsRBFA. Reactions were incubated at 37°C for 30 min, mixed with loading buffer which contained 40% sucrose and then resolved on native 6% 19:1 polyacrylamide gels using 0.5× Trisborate/EDTA as running buffer at 120 V for 20 min. The dsRNA gels were stained by gel-red and scanned using GenoSens 2000 (Clinx Corp.), and ssRNA Gels were scanned using a Typhoon FLA 7000 instrument (GE Healthcare). Each experiment was performed in triplicate.

### Fluorescence polarization (FP)

Lyophilized 5'-FAM-labeled RNA oligomers were purchased from Accurate Biology Inc. (Hunan, China). Experiments were performed in 200 μl volumes of buffer D (20 mM Tris–HCl, pH 8.0, containing 200 mM sodium chloride) containing 40 nM RNA and increasing concentrations of hsRBFA. Each RNA run was performed in parallel in three sets of experiments, and each set of experiments included 12 protein concentrations. Fluorescence polarization values (excitation) were measured using Spectra Max M5 (Molecular Devices) at a wavelength of 486 nm and an emission wavelength of 525 nm.

### Cell lines

HeLa, HepG2 (Shanghai Qincheng Biotechnology, Cat#QC163) and HEK293T cells were cultured in Dulbecco's modified Eagle's medium supplemented with 10% (v/v) fetal bovine serum and 1% penicillin/streptomycin. Cells were grown at 37°C in a humidified atmosphere containing 5% CO_2_. Trypan blue exclusion was used to assess cell viability.

### Plasmids and stable cell lines

All short hairpin RNAs (shRNAs) (sequences were reported before (22), Supplementary Table S2) against hsRBFA were inserted into PLKO.1 vectors (Huayueyang Bio., Beijing, China, Cat#VECT76534). Cells were infected with lentivirus followed by antibiotic selection to establish stable cell lines followed the protocol adopted from previous (50).

### Western blotting

Cell lysates were prepared in radioimmunoprecipitation assay buffer (50 mM Tris–HCl, pH 8.0, containing 150 mM NaCl, 5 mM EDTA, 0.1% SDS and 1% NP-40) supplemented with protease inhibitor cocktails (Roche). Equal amounts of total cell lysate were separated by SDS-PAGE. β-Tubulin served as a loading control. Horseradish peroxidase-conjugated anti-rabbit or anti-mouse (Bio-Rad) secondary antibodies were used to detect primary antibody binding (Respective primary antibodies, listed in Supplementary Table S3), and the signal was detected using LAS4000mini (GE bio. Inc.).

### Real-time fluorescence quantitative PCR (Q-PCR)

TRIzol (Invitrogen, Cat#15596018) extracted total RNA from HeLa cells and reverse transcribed it into cDNA for real-time fluorescence quantitative PCR. SYBR Green IWe fluorescent dye and a Roche LC96P temperature gradient fluorescence quantitative PCR instrument were used, with GAPDH as an internal reference (primer sequence shown in Supplementary Table S4).

### Mitochondrial respiratory and glycolysis activity assay

Mitochondrial respiratory and glycolysis activity in HeLa cells were measured by using the Seahorse Extracellular Flux Analyzer XFp (Agilent Technologies) with the XF Cell Mito Stress Test Kit (Agilent Technologies). Transfected cells (1 × 10^4^) were counted using ADAM-MC2 (NanoEntek) and plated into V3-PS 96-well plates the day before performing the assay. Standard mitochondrial stress tests were performed by first measuring basal values followed by measurements after sequential addition of 1 μM oligomycin, 1.25 μM FCCP and 0.5 μM rotenone/antimycin A. Glycolysis Stress Test performed by first measuring basal values followed by measurements after sequential addition of 10 mM glucose, 1 μM oligomycin, and 50 mM 2-DG (2-deoxy-glucose).

### Reactive oxygen species (ROS) and membrane potential detection

MitoSOX (Yeasen, Cat#40778ES50) and DCFDA/H2DCFDA dye (Abcam, Cat#ab113851) were used for the determination of mitochondrial and cellular reactive oxygen species. TMRM (Abcam, Cat#275547) was used for the determination of membrane potential. The corresponding channels were detected by flow cytometry.

## RESULTS

### hsRBFA interacts with 12S rRNA through its N-terminus and KH-like domain

As mentioned previously, hsRBFA bound with 12S rRNA through the region that mainly contained helix 44 and helix 45 using cross-linking immunoprecipitation (CLIP) assays (22,48). hsRBFA contains a classical KH-like domain, which contains a folding similar to that of its prokaryotic homologs (Figure 1B and Supplementary Figure S1a). Therefore, we first checked the binding between the hsRBFA KH-like domain (residues from Arg^86^ to Asp^201^, visualized in Figure 1A) and different RNA fragments using the electrophoretic mobility shift assay (EMSA). Surprisingly, we found that none of helix 28, helix 44, helix 45, or the single-strand 3′-terminal end can interact with the hsRBFA KH-like domain (Supplementary Figure S1b). However, when the KH-like domain of hsRBFA was extended (Nt-KH; Lys^42^ to Asp^201^, containing N-terminus and KH), it could bind with helix 44, helix 45 and helix 28 (Figure 1C). To further understand whether the C-terminus (Ct) of hsRBFA contributed to RNA recognition, we analyzed the substrate binding abilities of the near full-length (Lys^42^ to Glu^343^) and KH-like plus Ct (KH-Ct; Arg^86^ to Glu^343^) residues of hsRBFA. As expected, the near full-length hsRBFA bound with substrate RNAs at a similar level as Nt-KH hsRBFA (Figure 1D). KH-Ct cannot bind with any RNA substrates due to the lack of Nt (Figure 1E). Furthermore, we also purified the Nt of hsRBFA alone and noticed that this region can bind with helix 44, helix 45 and helix 28, but the binding pattern was wired. The hsRBFA–RNA complex formed a super-complex/glue-like complex, precipitated in the well and could not run into the polyacrylamide gel when we carried out the EMSA (Figure 1F). Therefore, hsRBFA bound to 12S rRNA through both the KH-like domain and the Nt, and Nt was the major element.

### hsRBFA binds to the double-stranded RNA of 12S rRNA instead of single-stranded RNA

**Figure 2.**
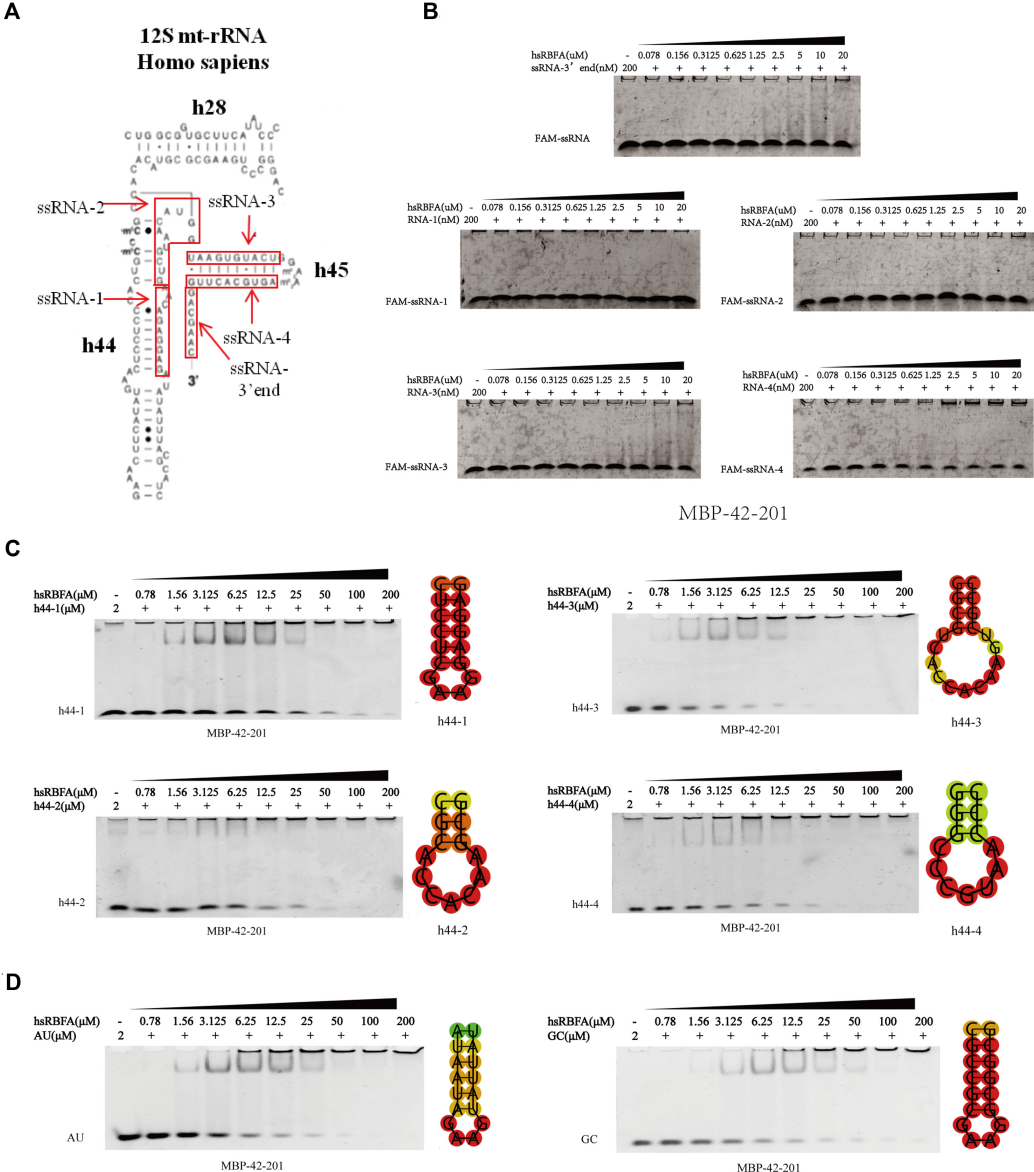
hsRBFA binds to the double-stranded RNA of 12S rRNA instead of single-stranded RNA. (**A**) Diagram visualizing the different regions of human 12S mt-rRNA. (**B**) EMSA of the binding affinity between different single-stranded RNAs (ssRNAs) of 12S rRNA and Nt-KH domains of hsRBFA. (**C**) EMSA results of the affinity between different lengths of helix 44 double-stranded RNA (dsRNA) and Nt-KH domains of hsRBFA. (**D**) EMSA results of the affinity between different artificial dsRNA fragments (randomly arranged AU and GC pairs) and Nt-KH domains of hsRBFA.

To better understand the binding details between the hsRBFA and its RNA substrate, we also characterized the RNA nature in the hsRBFA–RNA complex. The RNA fragments we used before contained not only the single strand regions but also the stem-loop regions, so we checked whether the hsRBFA primarily bound with single strand RNA and double-strand RNA first. We chose several variants of the stem-loop of helix 44 and carried out EMSA with hsRBFA Nt-KH protein. From the results, we noticed that hsRBFA can interact with any one of them (Figure 2C and Supplementary Table S1). At the same time, we picked up several single-stranded sequences from helix 44 to the 3′ end region of 12S rRNA (Figure 2A), and the EMSA showed that none of the single strand RNA derived from 12S rRNA can interact hsRBFA (Figure 2B, Supplementary Figure S2a and Supplementary Table S1). These results indicated that hsRBFA only interacted with double-stranded RNAs (dsRNAs) but not single-stranded RNAs (ssRNAs). To further investigate whether the hsRBFA recognized only the 12S rRNA sources dsRNAs or any sequences, we synthesized artificial dsRNA fragments. The EMSA results showed that both AU-rich RNA and GC-rich random RNA can be recognized by hsRBFA (Figure 2D). Astonishingly, we found that hsRBFA can also be complexed with dsDNA, as shown in Supplementary Figure S2b. Comprehensively, we also checked whether the KH-like domain alone of hsRBFA may have a different binding pattern from before. The results of EMSA between different ssRNAs with KH-like hsRBFA showed that none of ssRNA bound with hsRBFA KH-like. Taken together, we concluded that the Nt-KH of hsRBFA can only bind with dsRNAs but has no sequence specificity.

### Among the N-termini of hsRBFA, the basic amino acids patterned the interface of the hsRBFA–12S rRNA complex

**Figure 3.**
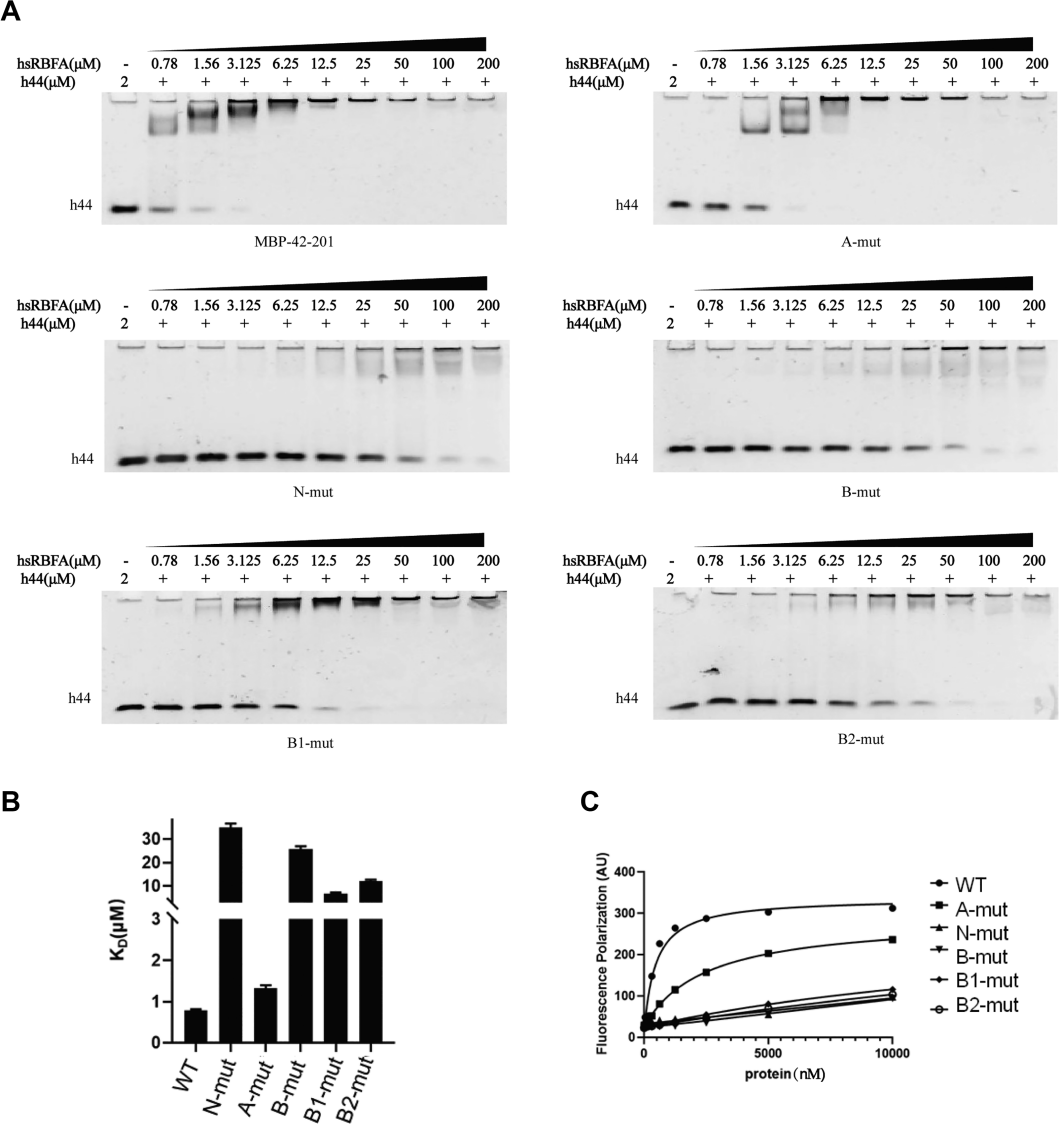
Among the N-termini of hsRBFA, the basic amino acids patterned the interface of the hsRBFA–12S rRNA complex. (**A**) EMSA results of the affinity between helix 44 of 12S rRNA and different mutations on the N-terminus of hsRBFA. (**B**) Comparison of fitting KD values of mutants. (**C**) FP analyzing the affinity between helix 44 of 12S rRNA and different mutations of hsRBFA.

Since Nt of hsRBFA contributes more to the interaction between hsRBFA and 12S rRNA, we wanted to analyze the molecular details of how Nt of hsRBFA bound with dsRNA fragments. We analyzed the amino acid sequences of its Nt domain and noticed that there were four negatively charged amino acids and nine positively charged amino acids among 48 residues in this region. hsRBFA recognizes dsRNA with no sequence distinction, so we predicted that only the basic residues would contribute to the interactions. Based on this hypothesis, we made three mutations: A-mut (mutation of all 4 acidic residues to alanines), B-mut (mutation of all 9 basic residues to alanines) and N-mut (mutation of all 13 amino acids in A-mut and B-mut). The results of the RNA binding experiments clearly showed that mutation of the positive charged amino acids abolished the RNA binding abilities, but the negative charged residues did not influence dsRNA binding (Figure 3A and B). After that, we further separated the nine basic residues into two subgroups: the B1 group (lysines 1st, 5th, 6th and 10th) and the B2 group (lysines 10th, 13th, 14th, 29th and 32nd) and mutated them into alanines. Interestingly, the EMSA indicated that both subgroups were important for hsRBFA to maintain binding with dsRNAs. Mutation of either subgroup partially abrogated dsRNA binding (Figure 3A and B). To confirm the authenticity of the results, the FP assay (fluorescence polarization) was used to quantitatively analyze the differences in dsRNA binding affinity (Figure 3C), and the results were consistent with the EMSA results. Therefore, it is reasonable to think that the binding of hsRBFA with 12S rRNA mainly depends on the basic amino acid residues on its N-terminus. On the other hand, we also carried out CD (circular dichroism) experiments with all different mutants to prove that all the mutants retained folding similar to that of the wild type (Supplementary Figure S2c).

### hsRBFA deficiency suppresses mitochondrial protein translation and impairs mitochondrial functions

**Figure 4.**
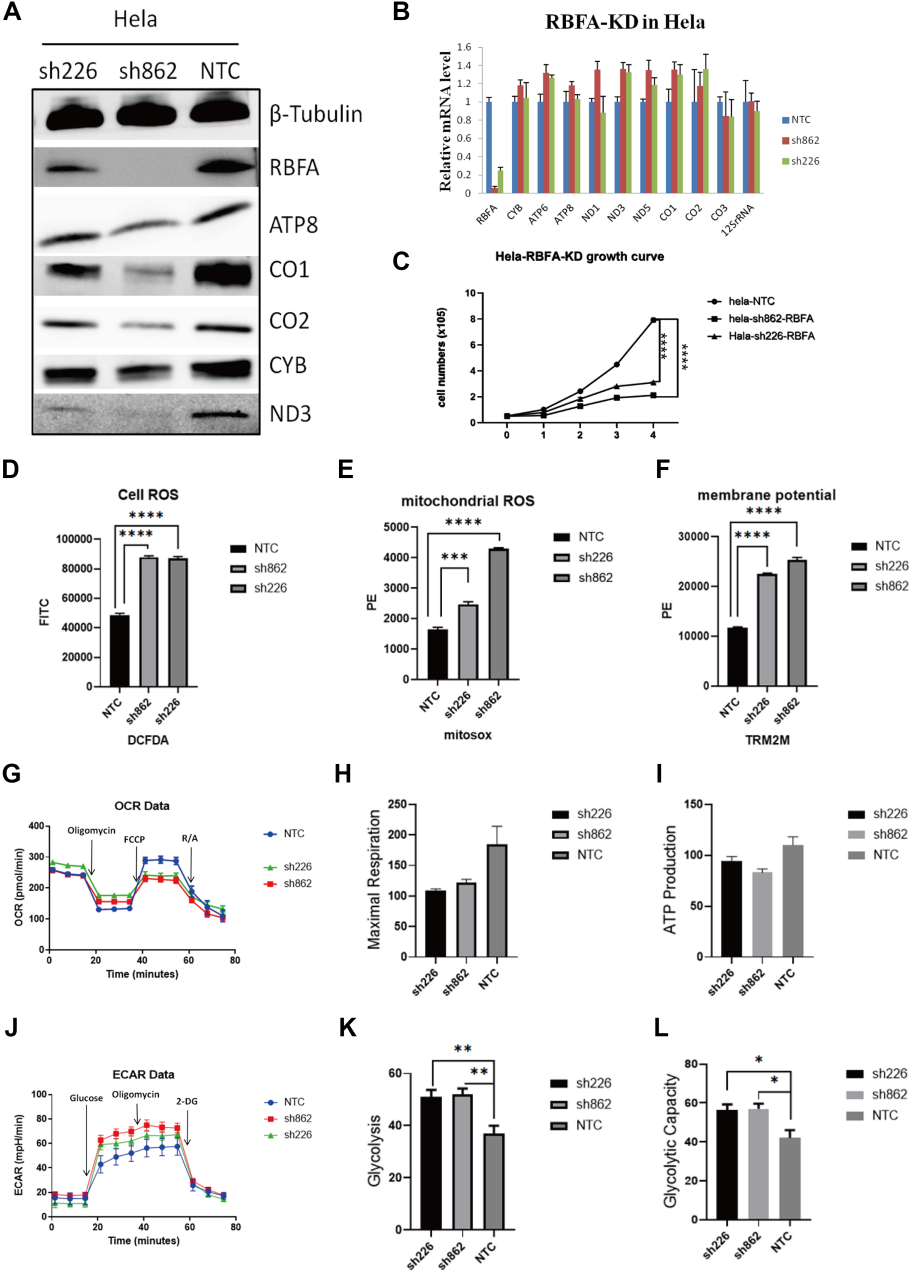
hsRBFA deficiency suppresses mitochondrial protein translation and impairs mitochondrial functions. (**A**) Western blot showing the protein levels of hsRBFA and other OXPHOS complex components coded by mitochondrial DNA in different cell lines (sh226 and sh862: HeLa cells transfected with different shRNAs; NTC: HeLa cells transfected with nonspecific targeting shRNA control; all panels in this figure are labeled the same). (**B**) Quantitative real-time PCR analysis indicated the mRNA transcriptional levels encoding mitochondrial polypeptides in different HeLa cells (**C**). The growth curves of different HeLa cells. (**D–F**) Flow cytometry was used to analyze cellular reactive oxygen species, mitochondrial reactive oxygen species and membrane potential. (**G–I**) The oxidative phosphorylation (OCR) levels of different cells using the Seahorse Extracellular Flux Analyzer XFp. (**J–L**) The glycolysis (ECAR) levels of mitochondria in different cells using Seahorse Extracellular Flux Analyzer XFp.

As we understood the major binding characteristics between hsRBFA and 12S rRNA *in vitro*, we tried to further study the biological function of N-termini of hsRBFA on mitochondria in the cell. We knocked down hsRBFA in HeLa cells using two different shRNAs (sh226 and sh862, sequences in Supplementary Table S2). From the western blot results (Figure 4A), both shRNAs suppressed the hsRBFA protein expression level at least 60%, especially sh862, which eliminated almost 100% of the hsRBFA in the cell. After determining the knockdown efficiency, we checked the impact on the transcribing and translating levels of the genes, which were coded by mitochondrial DNA and key components of OXPHOS complexes, in the stable HeLa cell lines bearing hsRBFA knockdown. The quantitative real-time PCR results indicated that the mRNA levels of these genes did not change much (Figure 4B); however, the western blotting results were more significant. The protein levels of MT-ATP-8 (ATP synthase protein 8; complex V), MT-ND3 (NADH-ubiquinone oxidoreductase chain 3; complex I), MT-CYB (cytochrome b; complex III), MT-CO1 (cytochrome *c* oxidase I, complex IV) and MT-CO2 (cytochrome *c* oxidase 2; complex IV) were analyzed by western blotting with the corresponding antibodies. The results suggested that the expression of these proteins was obviously decreased due to the knockdown of hsRBFA gene in HeLa cells (Figure 4A). Therefore, the consequences of the suppression of hsRBFA impaired the translation, rather than transcription, of mitochondrial genes involved in OXPHOS. Moreover, the proliferation of HeLa cells with hsRBFA knockdown was also markedly suppressed (Figure 4C). To confirm this repression is not cell-type dependent, we also carried out the knockdown experiments using HEK293T and HepG2 cells. The expressions of the proteins coded by the mitochondria in these two cell lines were reduced the same as those in the HeLa cell, although the effects were not as significant as HeLa (Supplementary Figure S3a–c). Especially for the HEK293T cell line, the influence of depleting hsRBFA can only be observed after 7 days. There was no reduction of mitochondria-coding protein levels after knocking down hsRBFA in HEK293T cells for 3 days, shown in Supplementary Figure S3d. This was similar to the previously reported results (22), which may be due to the turnover of ribosomes being slower in the first 3 days since they were matured (51). So we chose the HeLa cells for the following *in vivo* experiments. The malfunction of the OXPHOS complex influenced the proton pump gradients across the inner mitochondrial membrane and the balance of the ROS (reactive oxygen species) levels, so we monitored the mitochondrial membrane potential and ROS levels by flow cytometry. The results showed that both the membrane potential and ROS were increased when hsRBFA was knocked down in HeLa cells (Figure 4D–F and Supplementary Figure S4).

One major function of mitochondria is to provide energy through oxidative phosphorylation (OXPHOS) and glycolysis. Therefore, we also detected mitochondrial oxidative phosphorylation and glycolysis levels by Seahorse real-time cell metabolic analysis in HeLa cells under hsRBFA knockdown conditions. Although the oxidative phosphorylation level only decreased by ∼25%, the glycolysis level was significantly increased (Figure 4G–L). These results demonstrated that hsRBFA played an important role in maintaining mitochondrial function.

**Figure 5.**
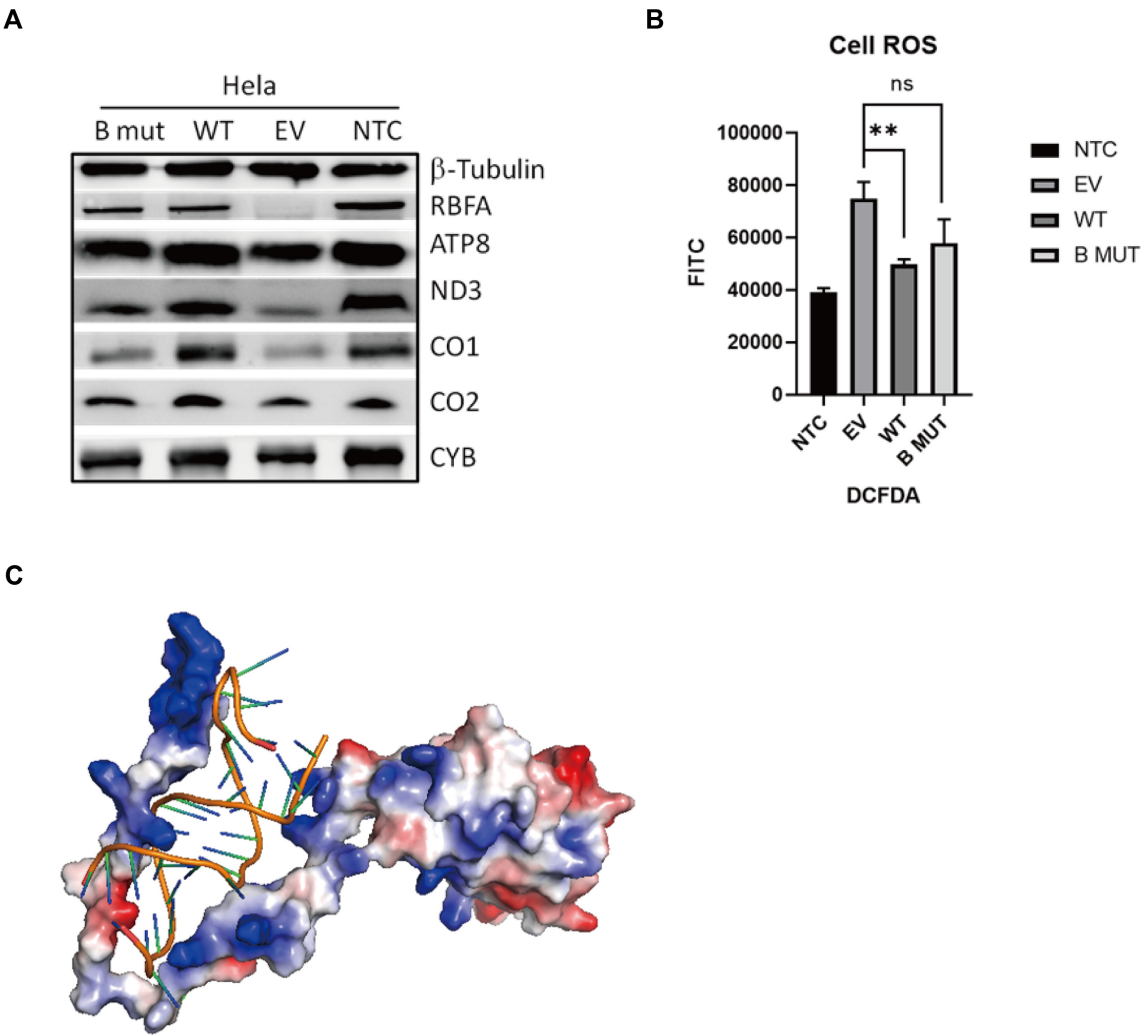
The N-terminal basic amino acids of hsRBFA play an important role in the maintenance of mitochondrial functions. (**A**) The protein expression levels of hsRBFA and mitochondrial gene-encoded proteins when overexpressing wild-type or N-terminal mutant hsRBFA (B mut as mentioned before) in HeLa cells with knocked down hsRBFA protein. (**B**) Flow cytometry revised cellular reactive oxygen species levels in HeLa cells overexpressing wild-type or N-terminal mutant hsRBFA. (**C**) Structure mode of the hsRBFA and helix 28 complex generated by HADDOCK Docking (v2.2) server based on the binding information we got (https://alcazar.science.uu.nl/services/HADDOCK2.2/haddock.php).

To confirm the effect of the N-terminus of hsRBFA defects *in vivo*, we overexpressed the wild type and B-mut of hsRBFA in HeLa cells that already knocked down endogenous hsRBFA using shRNA and checked the phenotypes. Western blotting experiments demonstrated that the expression levels of OXPHOS proteins were rescued by overexpressing wild-type hsRBFA but could not recover to similar levels in the B-mut-expressing group (Figure 5A). Consistently, cellular ROS analysis revealed that expressing wild-type hsRBFA, but not B-mut, promoted cellular ROS production to the relevant level as the non-suppressed group (Figure 5B).

Finally, by combining all these data, we suggested that hsRBFA interacted with its RNA substrate through its Nt-KH domain, employing a novel binding pattern. The N-termini behaved as the thumb, KH-like was the palm part of the hand, and they worked together to clamp the dsRNA regions of 12S rRNA as a hand (Figure 5C). The hsRBFA–12S rRNA complex acted as a platform to allow other proteins, such as TFB1M and METTL15, to load on, modify the 12S rRNA and promote the SSU of mitochondrial ribosome proper assembly and maturation. Furthermore, the correct function of hsRBFA is critical to the homeostasis of mitochondria and the whole cell.

## DISCUSSION

The bacterial RBFA protein has a type II KH-like domain, which can bind the 3′ end of 16S rRNA in the small subunit of the bacterial ribosome. Our study provides a new binding model of hsRBFA to the 12S rRNA model. The classical KH domain bound with ssDNA/ssRNA substrates through a GxxG motif, and the RBFA of *E. coli* also interacted with the single strand 3′ end region of 16S rRNA through its KH-like domain. The KH-like of RBFA from *E. coli* contained a conserved AxG motif (which is similar to the GxxG motif), but the hsRBFA did not have the motif. This difference may lead to different substrate recognition even though hsRBFA and other prokaryotic RBFA proteins share similar folding.

Yuzuru Itoh *et al.* recently reported the mitoribosomal SSU maturation process and mechanism (23). In the article, the authors mentioned that hsRBFA was recruited as a scaffold protein to the 12S rRNA encoding region, and then have a conformation change to adapt to the conformation of the small subunit assembly of the mitochondrial ribosome. Then, hsRBFA recruits and promotes METTL15 to methylate 12S rRNA. Meanwhile, in the CryoEM structures mentioned in their article, hsRBFA bound with the 12S rRNA near the 3′ end region, but may be due to the flexibility, the N-terminal information was not clear. However, from our results, we noticed that hsRBFA did not bind with the 3′ end ssRNA of 12S rRNA, so we expected that hsRBFA loaded to the position by the interaction between the hsRBFA Nt domain and stem−loop in helix 44 of 12S rRNA, not by the 3′ terminus ssRNA region. After the hsRBFA is loaded onto the mitoribosomal 28S pre-SSU (small subunit), the KH-like domain of hsRBFA may move close to the 3′ end region and be prepared for the following steps. On the other hand, we investigated the binding behavior of hsRBFA and dsRNA in detail and systematically both *in vitro* and *in vivo*. During our research, we noticed that the Nt is the key determinant of whether hsRBFA can bind with its substrate or not, and we did not notice the conformational change that Yuzuru observed in the CryoEM in the postmodificational steps. Therefore, we proposed that the conformational change may need some other protein help but may not be essential for the function of hsRBFA during it loaded to pre-SSU.

In conclusion, even though there are still some missing pieces, we carefully examined the mechanism of how hsRBFA recognized the dsRNA loops of 12S rRNA by its Nt-KH domain and reported a novel bound model of the KH family proteins. In addition, we examined the effect of disrupting the binding between hsRBFA and 12S rRNA on mitochondrial functions and cell homeostasis. Furthermore, we need to determine how hsRBFA helps other mitoribosomal maturating factors, such as TFB1M and METTL15, to be effective and promote the proper assembly of mitoribosomes and map a comprehensive map of how mitoribosomes become mature.

## DATA AVAILABILITY

The data underlying this article are available in the article and in its online supplementary material.

## Supplementary Material

gkac1234_Supplemental_File
