# Supplementary material for: The recognition mode between hsRBFA and mitoribosome 12S rRNA during mitoribosomal biogenesis
Source: Nucleic Acids Res. 2023 Jan 9;51(3):1353–63. doi: 10.1093/nar/gkac1234 (PMC9943654; doi:10.1093/nar/gkac1234)
Supplement: gkac1234_Supplemental_File [file gkac1234_supplemental_file.pdf]

The recognition mode between hsRBFA and mitoribosome 12S rRNA during mitoribosomal biogenesis

Wanwan Zhou[1,2], Xiaodan Liu[1,2], Mengqi Lv[1,2], Yunyu Shi[1,2,*], Liang Zhang[1,2,*]

1. Hefei National Research Center for Cross disciplinary Science, School of Life Sciences, Division of Life Sciences and Medicine, University of Science and Technology of China, Hefei, Anhui 230027, P.R. China.
2. Ministry of Education Key Laboratory for Membraneless Organelles and Cellular Dynamics, University of Science & Technology of China, Hefei, China

*To whom correspondence should be addressed. Liang Zhang, Tel: +86 551 63600441; Fax: +86 551 63601 443; Email: zhangl99@ustc.edu.cn

Correspondence may also be addressed to Yunyu Shi. Tel: +86 551 63607464; Fax: +86 551 63601443; Email: yyshi@ustc.edu.cn

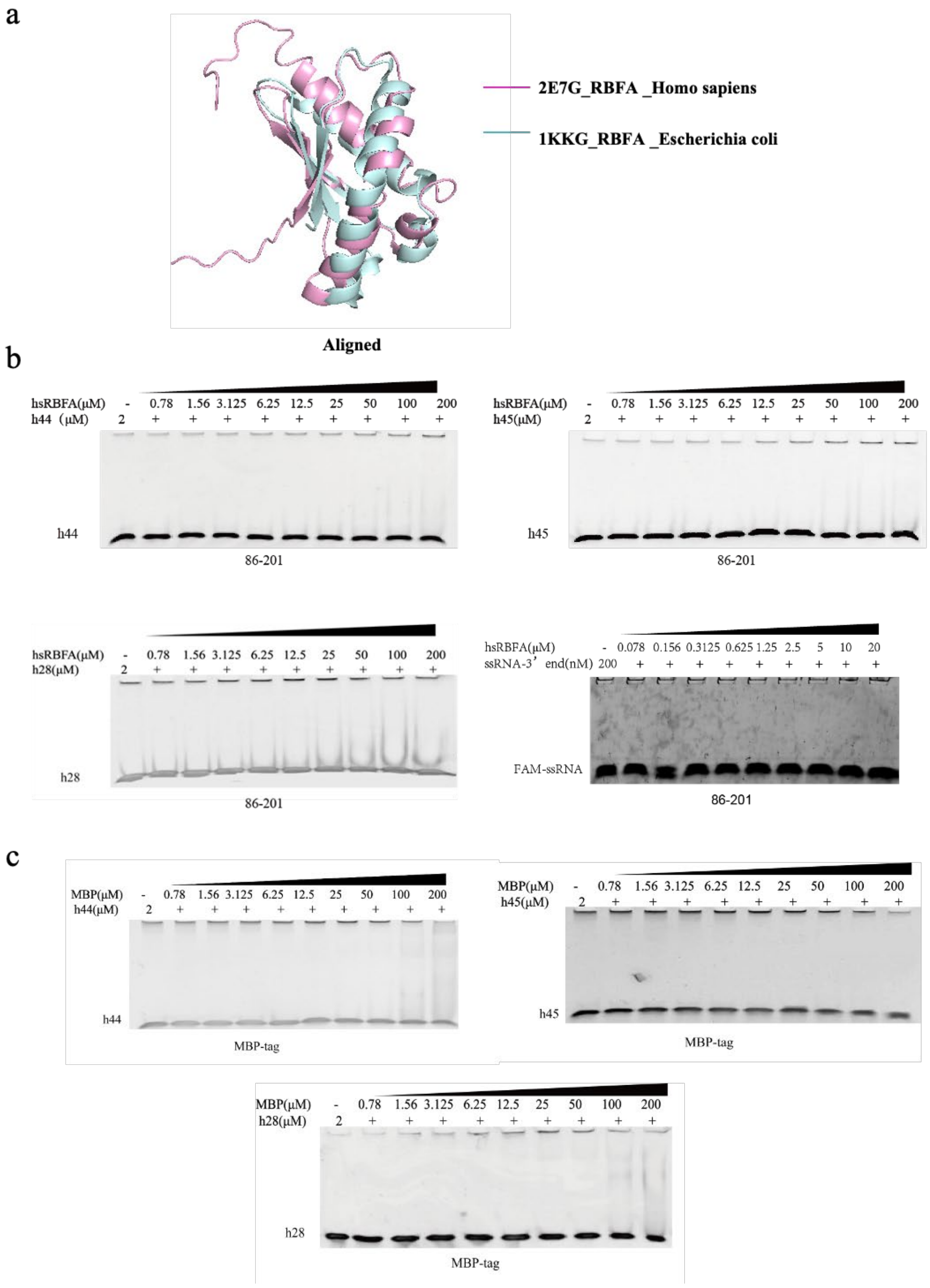

Figure S1. The KH-like domain of hsRBFA cannot interact with any RNA by itself. a. Structure alignment of KH-like domains of different homologous proteins. **b.** EMSA analysis of the interactions between the hsRBFA KH-like domain and helix 44, helix 45, helix28 or the 3’ end of 12S rRNA. **c.** EMSA results of the binding between helix 44, helix 45 or helix 28 and the MBP tag showed that the MBP tag did not bind with helix 44, helix 45 and helix 28.

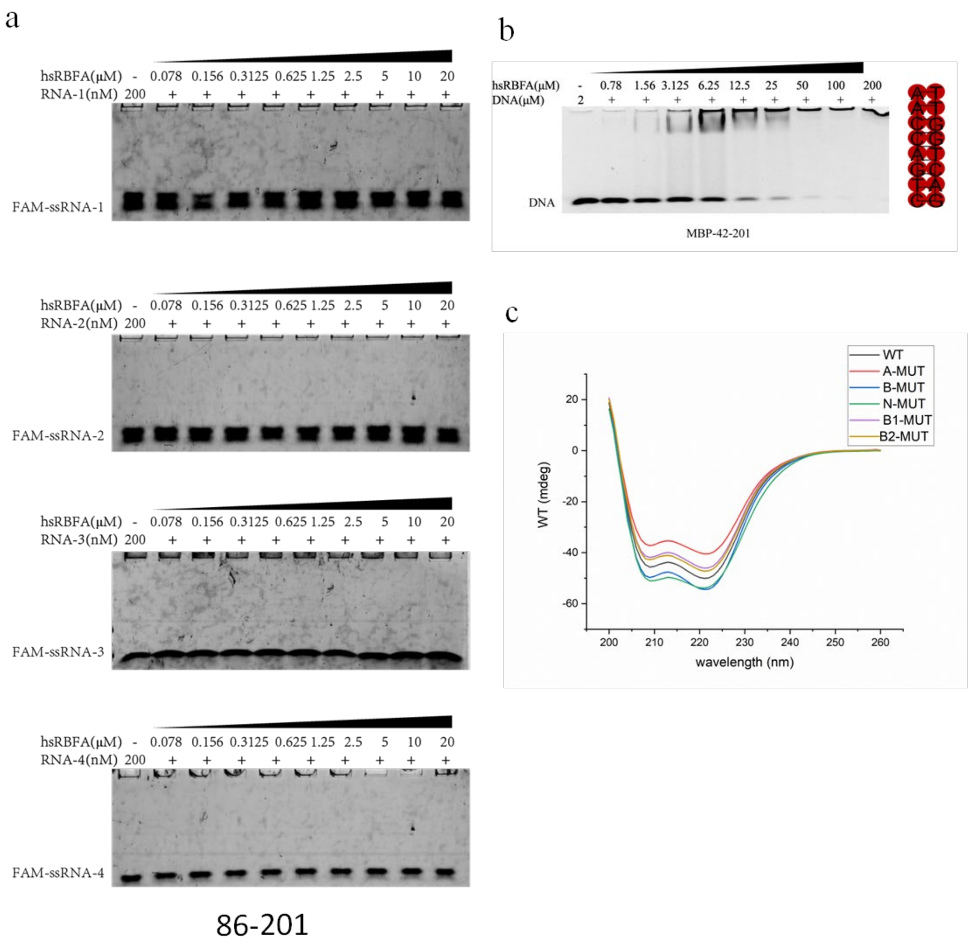

Figure S2. hsRBFA binds to any double-stranded RNA or DNA through Nt basic amino acids. a. EMSA indicated that the KH-like domain of hsRBFA did not interact with any single-stranded RNA. **b.** EMSA was used to analyze the binding affinity between dsDNA and Nt-KH domain of hsRBFA. **c. The** circular dichroism spectrum (CD) measured the secondary structures of different mutants, and the results revealed that there was no major difference among all the hsRBFA mutations.

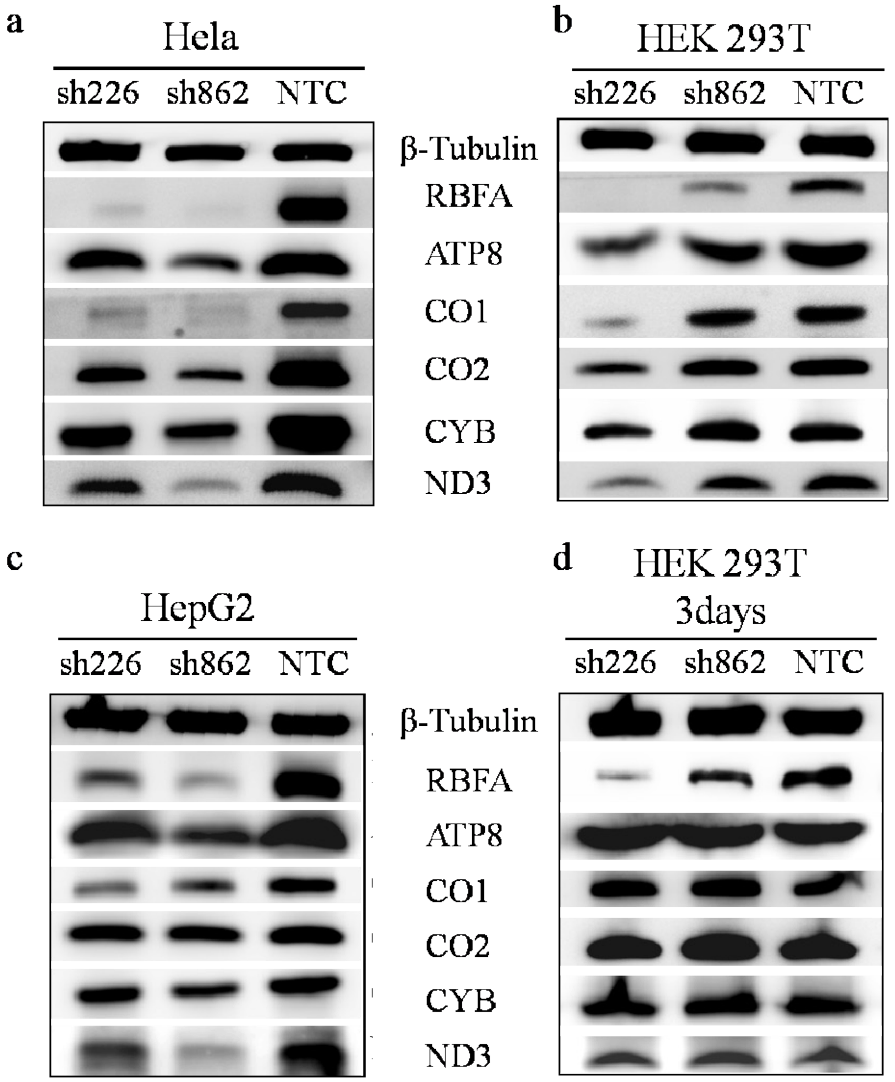

Figure S3. The expression levels of the mitochondrial gene encoding proteins after suppressing the hsRBFA in different cell lines. Western blot analyzing protein levels of hsRBFA and mitochondrial gene-encoded proteins in OXPHOS complexes in Hela cells **(a)**, HEK293T cells **(b)**, or HepG2 cells **(c)** treated with different RBFA shRNAs (sh226 or sh862) for 7 days. (d) The protein levels of hsRBFA and mitochondrial gene-encoded proteins in the HEK293T cells after shRNA treatment for only 3 days to analyze the short-term effects. NTC: cells transfected with nonspecific targeting shRNA control.

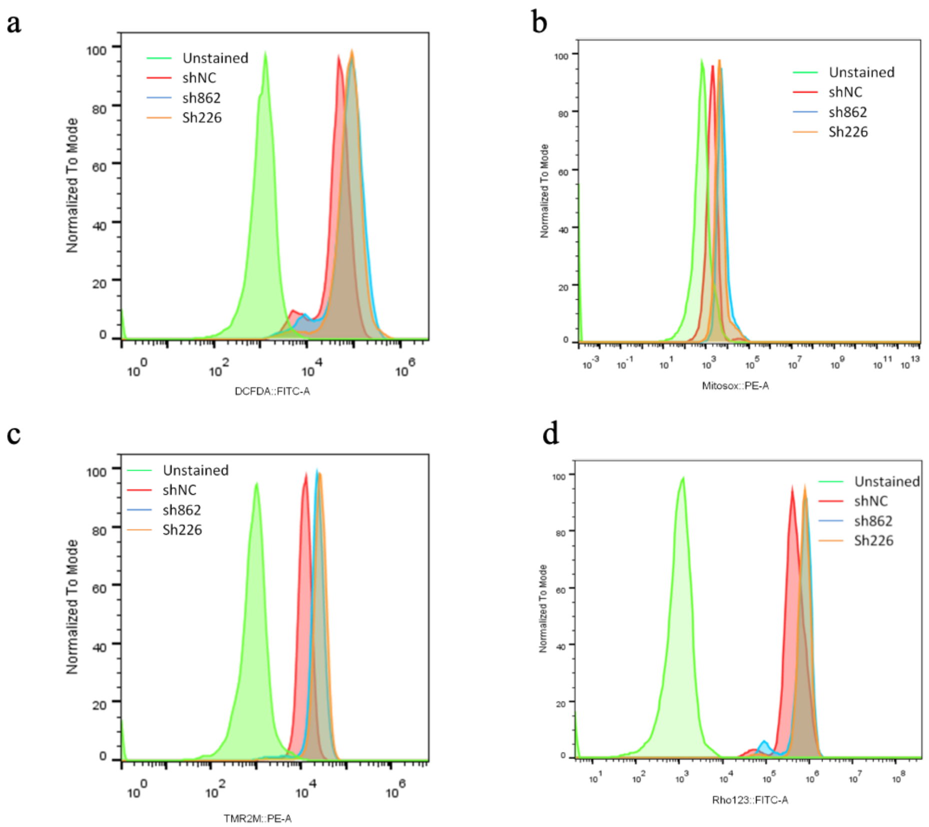

Figure S4. The membrane potential and ROS were increased when hsRBFA was knocked down in HeLa cells. Flow cytometry showed cellular reactive oxygen species (**a.** using DCFDA as a marker), mitochondrial reactive oxygen species **(b.** using MitoSOX as a marker), cellular membrane potential **(c.** using TRM2M as a marker) and cellular membrane potential **(d.** using Rho123 as a marker) in HeLa cells transfected with different shRNAs. (sh226 and sh862: HeLa cells transfected with different shRNAs; NTC nonspecific targeting shRNA control)

Table S1. RNA sequence used in this article

RNA name	RNA sequence
h44	GGCGUCACCCUCUUCGGAGACAAGUCGCC
h45	UAAGUGUACUGGAAAGUGCACUUG
h28	GGUGGCGGUGCUUCAUUCGUGAAGCGCGUCACC
AU	AUAAUAGAAGUAUUAU
GC	CGCCGCGAAGGCGGCG
DNA	TTGGTCAG/CTGACCAA
ssRNA-1	GAGGAGACAA
ssRNA-2	GUCGUAACAU
ssRNA-3	UAAGUGUACU
ssRNA-4	AGUGCACUUG
ssRNA-3'end	GACGAAC
h44-1	CUCCUCGAAGGAGGAG
h44-2	CGCACCACAAGCG
h44-3	GGCGUCACCACAAGUCGCC
h44-4	GGGCCCGUAACCC

Table S2. shRNA sequence used in this article

shRNA	sequence
sh226	GAACTGGCCAAGAAATTT
sh862	GGCGCTCAACAAGCAGATT

Table S3. **Antibody used in this article**

antibody	Company and article Number
Anti-β Tubulin	Abcam, T0023
Anti-RBFA	Thermo, pA5-59587
Anti-MT-CO2	Thermo, A-6404
Anti-MT-ND3	Abcam, ab192306
Anti-MT-CO1	Abcam, ab203912
Anti-MT-CYB	Abcam, ab219823
Anti-MT-ATP8	Proteintech, 26723-1-AP

Table S4. Q-PCR primer sequence used in this article

Q-pcr primer	sequence	
RBFA	Forward Sequence	AAGGCACTGACAGACCTGCTGT
	Reverse Sequence	TGTGTGCGTTCTGCTCAGCAGA
MT-CO2	Forward Sequence	CGTCTGAACTATCCTGCCCG
	Reverse Sequence	TGGTAAGGGAGGGATCGTT
MT-ND1	Forward Sequence	GGCTATATACAACTACGCAAAGGC
	Reverse Sequence	GGTAGATGTGGCGGGTTTTAGG
MT-CO1	Forward Sequence	CAAAGTATTTAGCTGACTCGCC
	Reverse Sequence	GAAAGATGAATCCTAGGGCTCA
MT-ATP6	Forward Sequence	CACAGTGATTATAGGCTTTCGC
	Reverse Sequence	GAGTAGGCTGATGGTTTCGATA
MT-ND3	Forward Sequence	ACAACTCAACGGCTACATAGAA
	Reverse Sequence	GTGGCAGGTTAGTTGTTTGTAG
MT-ND5	Forward Sequence	CTTCTAAACGCTAATCCAAGCC
	Reverse Sequence	TAGTGGGCTATTTTCTGCTAGG
MT-CYB	Forward Sequence	ATTATGGCTGAATCATCCGCTA
	Reverse Sequence	CAGAATGATATTTGGCCTCACG
MT-CO3	Forward Sequence	CCATAACGCTCCTCATACTAGG
	Reverse Sequence	GGTATGTGCTTTCTCGTGTTAC
MT-ATP8	Forward Sequence	TAAATACTACCGTATGGCCCAC
	Reverse Sequence	GTGATGAGGAATAGTGTAAGGAG
12S rRNA	Forward Sequence	CCCCAGGTTGGTCAATTTC
	Reverse Sequence	CGGCTTCTATGACTTGGGTTAA
